# Democratizing Public Health: Participatory Policymaking Institutions, Mosquito Control, and Zika in the Americas

**DOI:** 10.3390/tropicalmed8010038

**Published:** 2023-01-05

**Authors:** Michael Touchton, Brian Wampler

**Affiliations:** 1 Department of Political Science, University of Miami, Coral Gables, FL 33146, USA; 2 Faculty Lead for Global Health, Institute for Advanced Studies of the Americas, University of Miami, Coral Gables, FL 33146, USA; 3 President’s Office of Public Engagement, Boise State University, Boise, ID 83725, USA

**Keywords:** Zika, Brazil, Participation, health governance, health policy, Dengue

## Abstract

The Zika virus is a mosquito-borne virus spread primarily by *Aedes* mosquitoes. Zika cases have been detected throughout the mosquito’s range, with an epidemic occurring from 2015 to 2017 in Brazil. Many Zika cases are mild or asymptomatic, but infections in pregnant women can cause microcephaly in children, and a small percentage of cases result in Guillan–Barré syndrome. There is currently little systematic information surrounding the municipal spread of the Zika Virus in Brazil. This article uses coarsened exact matching with negative binomial estimation and ordinary least squares estimation to assess the determinants of Zika incidence across the ~280,000 cases confirmed and recorded by Brazil’s Ministry of Health in 2016 and 2017. These data come from Freedom of Information Act (FOIA) requests in Brazil and have not been published. We use data on the universe of individual Zika cases in Brazil and Geographic Information Systems (GIS) software to examine the virus at the municipal level across 5570 municipalities and construct a unique, unusually rich dataset covering daily Zika transmission. Additionally, our dataset includes corresponding local data on democratic governance, mosquito control efforts, and environmental conditions to estimate their relationship to Zika transmission. The results demonstrate that the presence of subnational democratic, participatory policymaking institutions and high levels of local state capacity are associated with low rates of Zika contraction. These models control for local healthcare spending and economic conditions, among other factors, that also influence Zika contraction rates. In turn, these findings provide a better understanding of what works for local health governance and mosquito control and makes important data public so that scholars and practitioners can perform their own analyses. Stronger models of Zika transmission will then inform mosquito abatement efforts across the Global South, as well as provide a blueprint for combatting Dengue fever, which is also transmitted by *Aedes* mosquitoes.

## 1. Introduction

The emergence and rapid spread of the Zika virus in the Americas represented a complex public health challenge at the time and a contemporary opportunity to learn about combating other pandemics through hindsight. From 2015 to 2018, over one million people tested positive for Zika infection in the Americas, with many more infections likely un-reported; Zika cases are often asymptomatic, diagnosis is difficult and easily confused with other diseases with similar symptoms, and access to testing is limited across the tropical regions of the Americas, where Zika is most prevalent [1,2,3]. Zika’s spread and its potential impact on newborns sparked a public health crisis and brief panic. The World Health Organization (WHO) removed Zika’s status as a Public Health Emergency of International Concern in 2017, but the disease remains prevalent throughout the Americas, with 31,451 cases recorded in 2021 in the Americas; Brazil has the highest cumulative incidence. Moreover, other arboviruses spread by *Aedes* mosquito species, such as Dengue Fever and Chikungunya, registered more than 2,500,000 cases in the Americas in 2021 [4,5,6].

Our research helps to combat Dengue, Chikungunya, and Zika by analyzing a large, unique database on Brazilian municipal governance and Zika transmission. Specifically, we constructed this database—the largest known database on Brazilian municipalities’ democratic governance, administrative capacity, and public health performance [7,8,9]—to better understand how local participatory policymaking institutions, local state capacity, and expert-designed social policies affect responses to public health challenges. Next, we used freedom of information requests in Brazil to compile and clean data on the universe of confirmed Zika cases for 2016 and 2017 in Brazil (280,000 cases in all [10,11,12,13]. These data are technically public, but completely inaccessible from a practical perspective and have not been used in published, peer-reviewed scholarship. In this article, we analyze these data to provide specific policy recommendations to control Zika’s spread, to manage other mosquito-borne illnesses, and to better understand the political and social determinants of public health.

Our central research question is as follows: Does the quality of local democratic governance influence public health? Scholars and practitioners expect democracy to improve public health by creating incentives for politicians to deliver public goods and mechanisms through which the public can hold politicians accountable [14,15,16,17]. Yet, it is not at all clear that these mechanisms translate to good governance, especially at the local level and for marginalized populations. The extent to which democracy improves public health for the poor in the Global South is particularly unclear because many studies focus on national-level analyses that obscure local variation in performance. Do local democratic institutions improve public health performance? Which ones and in what ways [7,18,19,20]? How governments answer these questions will inform healthcare and development policies that affect the lives of billions of people around the world. The results of our research will therefore aid policymakers, international funding agencies, civil society organizations, and academics to better understand the performance of local democratic institutions, community health policy, and social programs in general, not just for mosquito control.

Our overarching hypothesis is that Brazilian municipalities with more robust democratic institutions and state capacity to deliver services will have combatted Zika transmission better than municipalities without these institutions and capabilities. We find that the presence of subnational democratic, participatory policymaking institutions and high levels of local state capacity are indeed associated with systematically lower rates of Zika contraction, all else equal. Specifically, municipal environmental sanitation councils and mosquito control teams are both associated with low rates of municipal Zika transmission. Our models of Zika transmission control for local healthcare spending and economic conditions, among other factors, that also likely influenced Zika contraction rates [21,22]. The result is stronger models of *Aedes aegypti* mosquito abatement and public health programs that can inform government responses to Zika across Brazil and throughout the Global South.

Broadly, this study helps build knowledge to combat Zika from the novel perspective of local democratic governance. Our results support directly incorporating the public in policymaking by creating participatory policymaking institutions that capture local knowledge and leverage local energy every day, rather than every four years through elections alone. This finding is significant for governments, donor groups, public health advocates in civil society, and individuals because it identifies a pathway toward better mosquito abatement and public health performance through local institutional reform [23,24,25]. The first contribution this article makes is thus to evaluate the municipal determinants of arbovirus transmission and supply an institutional solution in the form of Brazil’s Environmental Sanitation Councils, which are relatively popular, cost-effective, ways to improve public health. Additionally, this study makes public important data on individual (anonymized) Zika cases so that scholars and practitioners can perform their own analyses to advance public health, in general, and combat Zika, Dengue, Chikungunya, and other mosquito-borne illnesses.

## 2. Background

Zika is a virus spread by the vector of *Aedes aegypti* mosquitoes. It can also be transmitted through sexual contact as well as through blood from mothers to children [12]. The Zika virus generates a range of symptoms, such as mild fevers and rashes, as well as asymptomatic cases. Zika can spread quickly, and its many asymptomatic cases make disease surveillance difficult. Zika’s more dangerous symptoms of microcephaly, birth defects, and encephalitis impact pregnant women and their unborn children disproportionately and can damage cognitive development over the life course [26,27]. Zika’s health impacts can be serious, and the disease’s long-term effects are not entirely clear [28,29,30,31]. Potentially serious health impacts coupled with a lack of vaccines or treatments make Zika dangerous, even if its WHO threat level has dropped since 2016 [32,33,34].

*Aedes aegypti* is an extremely adaptable mosquito, which has confounded community-based efforts to control or eradicate it [30,31,35]. Response capacity to the Zika pandemic varied considerably across countries. Lower-income countries and lower-income communities within those countries were disproportionately affected by the virus due to lack of resources for mosquito abatement, for prophylactic measures, such as screens or air conditioning, and for education [3,36,37].

Zika is a confounding virus from a conceptual standpoint as well: the virus is both transmitted by mosquitos and sexually transmitted. Like Dengue or Chikungunya, outbreaks are much likelier in dense, poor urban areas [3,36,37,38]. However, four out of five Zika cases may be asymptomatic, and the disease is rarely fatal for adults, which makes the disease less visible in literal and figurative senses for public health [39,40,41]. Instead, the most serious effects are on pregnant women and unborn fetuses, with months-long delays between infection and evidence of impact making it difficult to take real-time measures to control the virus’s spread. Moreover, adult female mosquitoes can pass Zika to their larvae, which allows the virus to survive even without a vector and without humans [42,43]. More like SARS or COVID-19, Zika’s appearance as a novel virus fostered its rapid spread among a population that had not previously been exposed [44]. Thus, the Zika virus unusually carries lessons for and may benefit from lessons drawn from Dengue, HIV [45], Ebola, and the COVID-19 pandemic [46].

## 3. Brazil’s Zika Response

The Zika epidemic of 2016–2017 hit Brazil harder than any country. The country’s 280,000 reported infections in our data rank it first in the region, though the mild nature of the disease, asymptomatic cases, and testing cross-reactivity with other viruses put unofficial case estimates in the tens of millions for Brazil [12]. There are several important factors that promoted Zika’s spread within Brazil. First, the seasonally wet, tropical climate in much of the country is conducive to *Aedes* breeding. Second, mosquito abatement efforts vary considerably across and within municipalities, with lower-income neighborhoods and municipalities featuring fewer abatement efforts, funding, or organization, on average. Standing water in these communities and dense populations without window screens or air conditioning provided ample opportunities for mosquito breeding and for contact with humans to transmit the disease [3,36,37,38].

Brazil’s national Zika response focused on three areas: vector control, access to healthcare for those afflicted with Zika-related symptoms, and research on the disease [47]. The first two parts of the national strategy included the use of community health teams already in existence through the Family Health Program (PSF) [48]. These municipal teams received additional federal funding in 2016 and 2017 to administer Zika test kits, distribute mosquito repellent throughout the community, and to focus on mosquito abatement for vector control on the one hand, and education to bring community members into Brazil’s national health system, the *Sistema Único de Saúde* (SUS), for treatment when necessary—especially for pregnant women [49].

The national response to Zika covered the entire country, but much of Brazil’s Zika response was decentralized, with municipalities and states also receiving increased health surveillance funds and increased responsibility for testing, tracing, and reporting to the Health Ministry [47,50,51,52]. However, many municipal governments lacked knowledge and resources to address basic issues surrounding mosquito abatement and lacked capacity for disease surveillance, even with extra funding. We thus focus on municipal variation in capacity and resources to respond to Zika across all of Brazil.

The decentralization of the vector control and health access pillars of Brazil’s Zika strategy intersected with ongoing challenges in poverty reduction, building state capacity, and an extremely divisive political crisis from the impeachment and removal from office of President Dilma Rouseff in 2016. For example, vector control and disease surveillance efforts increased dramatically in Rio de Janeiro, but focused on so-called “safe” areas, with low crime and low poverty [53]. Poor sanitation and waste management in low-income areas meant that many residents stored drinking water in barrels or improvised cisterns, which provided havens for breeding mosquitoes. Similarly, efforts to provide care to pregnant women infected with Zika were extensive among middle class and wealthy populations but omitted many poor neighborhoods [54,55,56]. Thus, poverty, capacity, and political support from state and national governments created a heterogeneous Zika response across Brazil’s 5570 municipalities [57].

### Can Extending Local Democracy Combat Zika?

Disparities in resources and capacity to confront public health challenges, such as the Zika epidemic, persist despite decades of representative democracy following the end of Brazil’s military dictatorship in the 1980s. Yet, experiments with direct citizen participation in decision-making, including for public health, have also emerged, and grown since the late 1980s [23,24,58]. These experiments take the form of participatory institutions: state sanctioned institutional processes that include both citizens and government officials. These institutions incorporate participation, deliberation, and oversight into Brazilian democracy and harness local knowledge and human capital to deliver better services. This new participatory governance may improve public health by empowering citizens, enhancing governance, and improving service provision.

Brazil’s public policy management councils are the most prevalent participatory institution in the country. There are more than 60,000 municipal councils across thematic issue areas, such as health, education, and women’s rights, and more than 300,000 citizens that hold elected positions on these councils [8]. Councils comprise, in equal parts, civil society representation and municipal government officials. Council members come from community groups, social movements, non-profit service providers, and unions [59]. Council members may propose new policies in thematic issue areas the council represents as well as approve or reject year-end budgets and government reports on compliance with legal frameworks [59,60]. These policy management councils thus hold formal veto power over spending in their issue area, the formal power to propose new spending, and the informal power of holding government officials to account through social and political pressure [23,24,58].

Increasing evidence points to policy management councils’ effectiveness in improving governance, service delivery, and outcomes across thematic areas as well as evidence supporting the use of participatory institutions in general [7,8,23,24,61,62].

Brazil’s federal government now provides fiscal incentives for municipal adoption of health, social assistance, children’s protection, education, and environment councils. Yet, there are at least 16 remaining councils (e.g., environmental sanitation, women’s councils, food security councils, etc.) that municipalities adopt “voluntarily” without federal incentives [61]. We focus on these voluntary policy management councils, especially environmental sanitation councils, as potentially relevant for executing Brazil’s Zika strategy at the municipal level.

The complex coordination problem required to execute the government’s Zika strategy at the municipal level demanded new techniques for building local administrative capacity as well as for partnering with civil society to carry out mosquito abatement, provide education about prophylaxis for mosquito-borne and sexually transmitted infections, extend disease surveillance, and improve access to healthcare for infected, vulnerable residents [63,64]. Almost all Brazilian municipalities already had health councils at the time of the Zika outbreak, but many fewer had environmental sanitation councils, which could theoretically coordinate and oversee the mosquito-abatement and education portion of the Zika strategy. In our data, 10% of municipalities had these councils in 2005, with the percentage increasing to 34% by 2016. The variation in the presence and the maturity of these institutions across Brazil’s municipalities leads to the following hypotheses:

**H_1_** **:** *Municipalities with environmental sanitation councils will have lower rates of Zika infection, all else equal.*

**H_2_** **:** *Municipalities that adopted environmental sanitation councils earlier will have lower rates of Zika infection than municipalities that adopted the institutions later, all else equal.*

## 4. Data and Methods

We test our hypotheses using Brazil’s municipalities as natural laboratories. Brazil had one of the earliest experiences with Zika in the most recent epidemic and collected more public data on infections than most other countries in the region. Moreover, Brazil has the broadest and deepest experience with participatory governance in the region, along with the simultaneous expansion of the state and economic growth for much of the decade preceding the epidemic. Studying Brazil thus allows us to capture large variation in institutions, programs, processes, and Zika rates across all 5570 municipalities to test our central hypotheses. We use these data to build models of Zika incidence at the municipal level, using coarsened exact matching with negative binomial estimation and ordinary least squares estimation. The results of estimation then demonstrate the extent to which statistically significant connections exist between environmental sanitation councils and other aspects of local Brazilian democracy and Zika incidence. All data transformation and analysis was performed in R, version 4.2.2 [65] and ArcGIS 10.8.2 [66].

Dependent Variable: Zika Cases per 100,000 Residents in Brazil’s 5570 municipalities, 2016–2017. These are daily, individual-level infection data from Brazil’s Ministry of Health [10], which we anonymized and aggregated to the municipal level. The mean is 41.17 cases per 100,000 residents and the standard deviation is 19.24.

Independent Variables: We begin with the annual presence of environmental sanitation councils at the municipal level. This indicator takes the form of a dummy variable, where a score of 1 reflects the presence of a council and 0 reflects its absence. We supplement this measure with the number of years that councils have been in place for municipalities that score a 1 on the first indicator. These data come from the Brazilian Institute for Geography and Statistics (IBGE) [67].

### 4.1. Family Health Program (PSF) Coverage

We capture information on a critical social program that targets low-income Brazilians and promotes preventive health: the Family Health Program. We expect this program to help combat Zika because it increases education surrounding mosquito-borne illnesses as well as STIs [17]. It also encourages poor families to seek basic health services through domicile health visits. Pregnant women and newborns are among the populations served; thus, the program is likely to influence Zika contraction and transmission for the most vulnerable populations. The Ministry of Health collects annual data on the percentage of eligible families that receive benefits from the PSF. The mean coverage level is 83% and the standard deviation is 28.

### 4.2. Local Administrative Capacity

Local administrative capacity is also likely to influence the quality of mosquito abatement programs, public health education, and delivery of health services. We capture variation in local administrative capacity through a measure of the quality of local management of the Bolsa Família program. Bolsa Família was a large conditional cash transfer program, with conditionalities focused on education and health—including sexual education and prevention of disease transmission through childbirth. It is administered at the municipal level and management quality varies considerably. The Ministry of Social Development (MDS) offers greater funding to cities that perform better on an annual Index of Decentralized Management (IGD). The quality of local management should therefore reflect a combination of local political commitment as well as existing municipal capacity. This variable is continuous from 0 to 1 (low to high); the mean score is 0.78 and the standard deviation is 0.16.

### 4.3. Municipal Healthcare Spending

We control for per capita municipal healthcare spending in our models of Zika incidence. We assess whether municipal healthcare spending influences municipal service provision and health outcomes. Brazil has relatively high public goods spending per capita, but low-quality health outcomes for the expenditure [59,68]. There is also a noted disparity in spending between more affluent and less affluent municipalities, which we expect to help explain variance in municipal rates of Zika contraction. The indicator is annual municipal health spending per capita, in constant Brazilian *Reais* (2010). The data for this indicator come from Brazil’s Health Ministry [10]. We use the base-10 logarithm of the raw values in our models.

### 4.4. Left Mayor

We control for the mayor’s political ideology to account for the possibility that those on the political left support healthcare and protections for the poor more than those on the right. These data come from Brazil’s Superior Electoral Tribunal.

### 4.5. Political Competition

We use the mayor’s vote share in the most recent election to control for the possibility that mayors who were eligible to run for re-election and won with smaller vote shares had incentives to take the Zika epidemic seriously prior to the 2016 municipal elections.

### 4.6. Geography and Seasonality

We control for the geographic region within Brazil and the month of the year to reflect seasonal and environmental trends encouraging mosquito breeding. Brazil’s South is the omitted category for geography.

## 5. Estimation Strategy

We use coarsened exact matching (CEM) as an identification strategy for causal inference. This strategy allows us to simulate a randomized controlled trial, with treatment and control groups, while still relying on observational data [69]. We pre-process the data using the R package “Matchit” [70] with CEM to assess whether municipalities that adopt environmental sanitation councils (the treatment) exhibit systematically different Zika incidence rates than municipalities that lack these participatory institutions (the control) but are as similar as possible regarding Family Health Program coverage, per capita health spending, local state capacity, political competition, the mayor’s political ideology, the month of the year, and geographic location within Brazil. This strategy is like propensity score matching, but with superior balance across covariates [69]. The result is a municipal comparison where the main observable difference across municipalities is the presence of an environmental sanitation council in one and its absence in the other.

We then estimate the relationship between environmental sanitation councils and Zika rates using conditional negative binomial models with municipal and year fixed effects. Negative binomial regression models are appropriate when count and rate outcome data are over-dispersed, as they are for Zika, where the unconditional mean is less than the variance [71] Column II uses OLS instead of negative binomial regression as a robustness check.

**Figure 1:**
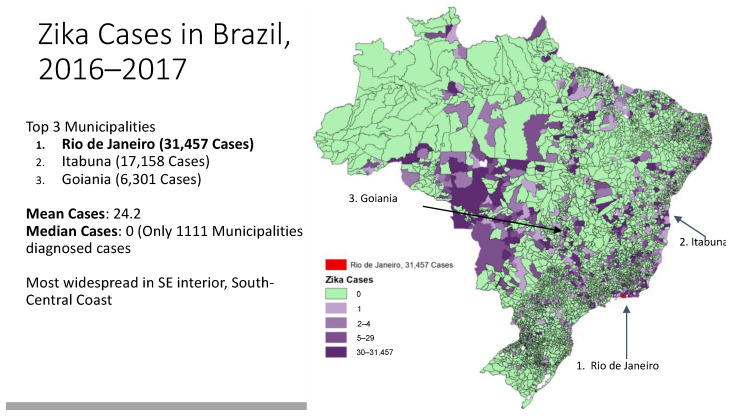
Zika Cases in Brazil, 2016–2017.

Figure 1 depicts the distribution of Zika cases across Brazil’s municipalities. As seen in the figure, cases are clustered in the Southeast and coastal regions of the country.

**Figure 2:**
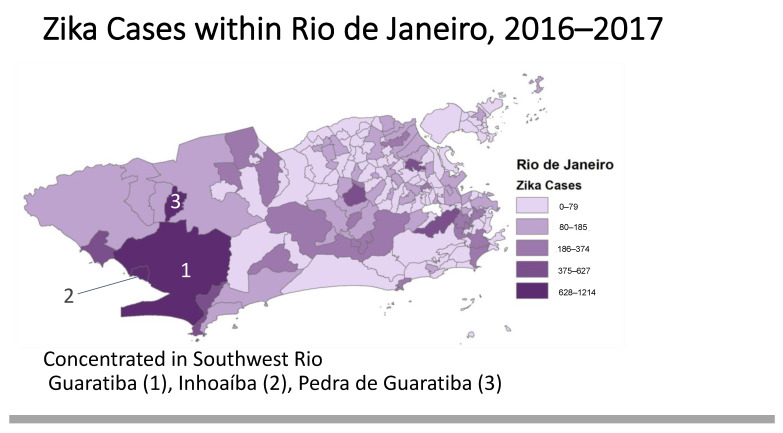
Zika Cases within Rio de Janeiro, 2016–2017.

Figure 2 showcases the sub-municipal distribution of raw Zika cases in one municipality, Rio de Janeiro, to demonstrate the granularity of our unique dataset and its added value for other researchers.

**Figure 3:**
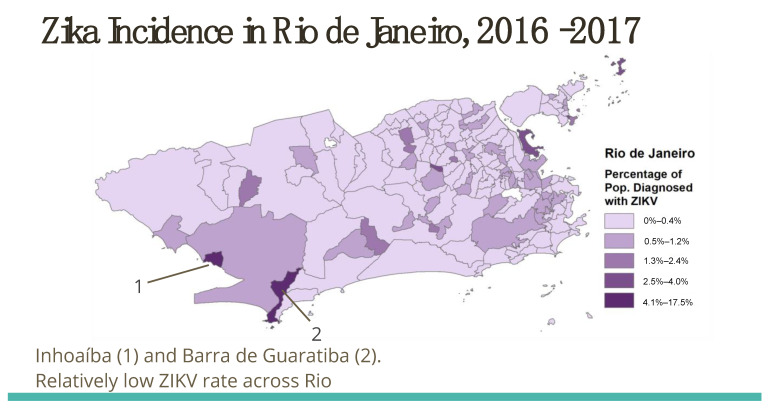
Zika Incidence in Rio de Janeiro, 2016–2017.

Figure 3 showcases the sub-municipal incidence of Zika cases in one municipality, Rio de Janeiro, to demonstrate the granularity of our unique dataset and its added value for other researchers.

**Figure 4:**
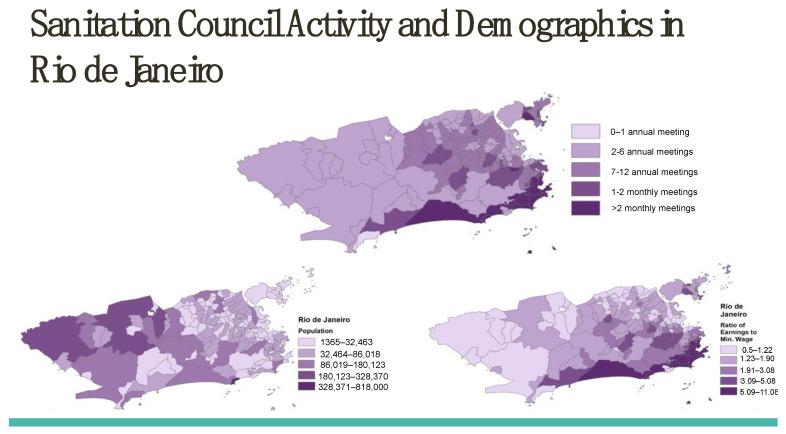
Sanitation Council Activity and Demographics in Rio de Janeiro.

Figure 4 presents Rio de Janeiro’s neighborhood-level distribution of environmental sanitation council activity and socioeconomic characteristics.

The distribution of active policy management councils maps onto Zika incidence, which then requires evaluation across the national landscape of Brazilian municipalities through matching and regression analysis. These results appear in Table 1, Table 2, Table 3 and Table 4 below.

## 6. Results and Discussion

The results of estimation demonstrate that the presence of a municipal environmental sanitation council is associated with 5.21 fewer Zika cases per 100,000 residents. On average, municipalities with environmental sanitation councils experience 12% fewer Zika cases (per 100,00 residents) than those that do not. Across Brazil, this translates to the potential for preventing approximately 34,000 Zika cases, if all municipalities had environmental sanitation councils, based on the universe of 280,000 reported cases in our data [10]. If higher estimates of tens of millions of mild and/or asymptomatic cases across Brazil are more accurate, then the equivalent estimate for prevented Zika cases rises to the low millions [39,40,41].

Even after pre-processing the data using Coarsened Exact Matching, greater municipal health spending, greater municipal coverage through the Family Health Program, and greater municipal state capacity are all associated with lower Zika incidence [72,73]. Municipalities’ geographic region is also important, with Southeastern and Northeastern cities experiencing greater Zika incidence, on average. Similarly, the months of January, February, March, and April have higher incidence than the omitted baseline month of July [74,75,76,77]. Tables with full geographic and seasonal variables will be posted along with the dataset in the Harvard Dataverse.

The length of time that environmental sanitation councils have been in place is also a statistically significant determinant of municipal Zika incidence [9,23,24,25]. We estimate that each additional year of use of the council is associated with 1.57 fewer Zika cases per 100,000 residents, on average. The longest use in the dataset is 16 years, which translates to a 61% lower Zika incidence in these municipalities, all else equal. Like with the models in Table 2, per capita municipal health spending, geographic, and seasonal variables remain statistically significant determinants of Zika incidence, even after pre-processing. These results suggest that adopting environmental sanitation councils might not immediately improve mosquito abatement and reduce Zika, Dengue, or Chikungunya. Institutional reforms need time to mature and might achieve their maximum potential only after considerable time in use [8,23,24,25].

We then explore interactions between environmental sanitation councils and other covariates in models with full variation across municipalities, rather than matching them on all observables. We find that interactions between the councils and municipal health spending, municipal state capacity, and family health program coverage all have negative, statistically significant effects on Zika incidence at the municipal level. These models are presented in Tables S1–S6 of the supplementary material and suggest that multiple aspects of local democratic governance, beyond the presence of environmental sanitation councils alone, can also affect Zika, in this case, and likely public health performance in general [7,8,23,24,25].

## 7. Limitations

Zika transmission varies considerably within municipalities, as does the policy response. For example, Rio de Janeiro had many Zika cases, but cases were heavily clustered in lower-income parts of the city, while many wealthier neighborhoods had few cases [78,79]. Our data on Zika cases are at the street and neighborhood level, but most governance and policy data are at the municipal level, which cannot explain the important sub-municipal variation in Zika transmission [8,80]. Explaining this variation is critical to inform policy responses and, put simply, cannot be done without corresponding, but currently unobtainable, data on neighborhood governance, health education, and mosquito control responses. Finally, there remains some possibility that municipalities that voluntarily adopt environmental sanitation councils are predisposed to better health governance or have other unobserved characteristics that might influence Zika incidence, such as a better educated or better engaged citizenry. Using coarsened exact matching to pre-process the data is designed to mitigate these concerns as much as possible; we only compare the most similar municipalities on Family Health Program coverage, per capita health spending, local state capacity, political competition, the mayor’s political ideology, the month of the year, and geographic location within Brazil to each other, with the observed difference being the presence or absence of environmental sanitation councils. The strategy makes it very unlikely that one municipality’s predilection toward good governance or health services, for example, would remain after being matched on the other dimensions.

## 8. Conclusions

Zika is a complex, unusual disease that demanded a multi-faceted response of vector control and improvements in public health access in Brazil [3,36,37,38]. Participatory policymaking institutions, such as environmental sanitation councils, enhanced this response in municipalities that used them by incorporating the public and civil society into the mosquito abatement, disease surveillance, and public health education efforts [18,19,20,25]. In general, our evidence suggests that promoting participatory institutions in Brazil and around the world may be justified to improve governance and public health. Moreover, our results demonstrate the relevance of other elements of governance for controlling Zika, including the administration of national health programs, such as the FHP, the capacity of the local government to administer programs in general, and the allocation of local funds to healthcare. These areas are not targeted toward Zika in any specific sense and are therefore potentially relevant for public health performance as well [23,24,25].

Public involvement in ongoing policymaking processes for Zika and other epidemics, should be prioritized to reduce health disparities and improve public health responses. Building capacity for co-governance in public health is an important step for addressing current and future public health challenges in effective, equitable manners. Further, collaboration across government and civil society may also help to increase local capacity to combat disease and deliver health services through community-based human capital [23,24,25]. Implementing multifaceted strategies to combat complex diseases, such as Zika, requires attention to social, economic, and political issues that go far beyond laboratory-based, purely medical conceptualizations of public health [21,22,29]. Empowering citizens and civil society through local participatory institutions can use robust local democracy to combat disease and promote better public health performance.

## Figures and Tables

**Table 1:** This table reports the results of negative binomial estimation following pre-processing through Coarsened Exact Matching.

Municipal Zika Cases per 100,000 Residents			
Predictors	Estimates	CI	*p*
(Intercept)	38.92	28.38–49.46	<0.01
Environmental Sanitation Council	−5.21	−4.68–−5.74	<0.01
FHP Coverage	−0.28	−0.14–−0.42	0.04
Local State Capacity	−0.18	−0.06–−0.33	0.17
Mayoral Vote Share	−0.27	0.24–−76	0.34
Left Mayor	0.35	0.10–0.53	0.23
Health Spending (per capita, logged)	−0.53	0.26–0.71	<0.01
Observations	5382		
Wald Chi^2^ (6)	239.42		
Prob > Chi^2^	0.00		
R^2^	0.34		

**Table 2:** This table reports the results of OLS estimation following pre-processing through Coarsened Exact Matching.

Municipal Zika Cases per 100,000 Residents			
Predictors	Estimates	CI	*p*
(Intercept)	40.10	27.94–51.38	<0.01
Environmental Sanitation Council	−5.04	−4.39–−5.61	<0.01
FHP Coverage	−0.31	−0.20–−0.39	0.02
Local State Capacity	−0.20	−0.09–−0.31	0.04
Mayoral Vote Share	−0.16	0.32–−86	0.43
Left Mayor	0.22	0.03–0.64	0.15
Health Spending (per capita, logged)	−0.29	−0.13–−0.44	<0.01
Observations	5264		
Wald Chi^2^ (6)	251.37		
Prob > Chi^2^	0.00		
R^2^	0.29		

**Table 3:** Maturity of Environmental Sanitation Policy Councils and Zika, 2016–2017. This table reports the results of negative binomial estimation following pre-processing through Coarsened Exact Matching for each year that municipalities used an environmental sanitation council.

Municipal Zika Cases per 100,000 Residents			
Predictors	Estimates	CI	*p*
(Intercept)	39.04	27.83–49.26	<0.01
Time with Environmental Sanitation Council	−1.57	−1.03–−2.11	<0.01
FHP Coverage	−0.16	0.05–−0.31	0.14
Local State Capacity	−0.09	0.10–−0.26	0.21
Mayoral Vote Share	−0.21	0.25–−66	0.38
Left Mayor	0.27	0.07–0.43	0.15
Health Spending (per capita, logged)	−0.45	−0.17–−0.62	<0.01
Observations	4959		
Wald Chi^2^ (6)	220.67		
Prob > Chi^2^	0.00		
R^2^	0.37		

**Table 4:** Maturity of Environmental Sanitation Policy Councils and Zika, 2016–2017. This table reports the results OLS estimation following pre-processing through Coarsened Exact Matching for each year that municipalities used an environmental sanitation council.

Municipal Zika Cases per 100,000 Residents			
Predictors	Estimates	CI	*p*
(Intercept)	41.23	24.27–56.35	<0.01
Time with Environmental Sanitation Council	−1.29	−1.05–−1.42	<0.01
FHP Coverage	−0.25	0.09–−0.46	0.24
Local State Capacity	−0.11	0.06–−0.23	0.16
Mayoral Vote Share	−0.20	0.29–−65	0.33
Left Mayor	0.24	−0.08–0.46	0.09
Health Spending (per capita, logged)	−0.17	−0.06–−0.27	<0.01
Observations	4955		
Wald Chi^2^ (6)	228.70		
Prob > Chi^2^	0.00		
R^2^	0.31		

## Data Availability

All data are available at: https://dataverse.harvard.edu/dataset.xhtml?persistentId=doi:10.7910/DVN/KDSF60, accessed on 13 May 2022.

## Supplementary Materials

The following supporting information can be downloaded at: https://www.mdpi.com/article/10.3390/tropicalmed8010038/s1, Table S1: Negative Binomial Regression with Interactions between councils and FHP Coverage. Table S2: OLS Regression with Interactions between councils and FHP Coverage. Table S3: Negative Binomial Regression with Interactions between councils and local state capacity. Table S4: OLS Regression with Interactions between councils and local state capacity. Table S5: Negative Binomial Regression with Interactions between councils and health spending. Table S6: OLS Regression with Interactions between councils and health spending.
