# Supplementary material for: Democratizing Public Health: Participatory Policymaking Institutions, Mosquito Control, and Zika in the Americas
Source: Trop Med Infect Dis. 2023 Jan 5;8(1):38. doi: 10.3390/tropicalmed8010038 (PMC9865320; doi:10.3390/tropicalmed8010038)
Supplement: Supplementary file 1 [file tropicalmed-08-00038-s001.zip › tropicalmed-2073087-supplementary.pdf]

Table S1: Negative Binomial Regression with Interactions between councils and FHP Coverage

| | | Municipal Zika Cases per 100,000 Residents | |
|---|---|---|---|
| Predictors | Estimates | CI | p |
| (Intercept) | 37.94 | 26.59 – 47.32 | <0.01 |
| Councils*FHP | -7.21 | -7.04- -7.66 | <0.01 |
| Environmental Sanitation Council | -0.56 | -0.21 – -1.09 | 0.38 |
| FHP Coverage | -0.10 | 0.19 – -0.63 | 0.22 |
| Local State Capacity | -0.87 | -0.66 – -1.17 | 0.04 |
| Mayoral Vote Share | -0.23 | 0.22 – -78 | 0.36 |
| Left Mayor | 0.37 | 0.11 – 0.51 | 0.27 |
| Health Spending (per capita, logged) | -0.95 | -0.75 – -1.22 | <0.01 |
| Observations | 5376 | | |
| Wald $Chi^2$ (6) | 231.67 | | |
| Prob> $Chi^2$ | 0.00 | | |
| $R^2$ | 0.38 | | |

Table S2 OLS Regression with Interactions between councils and FHP Coverage

| | Municipal Zika Cases per 100,000 Residents | | |
|---|---|---|---|
| Predictors | Estimates | CI | p |
| (Intercept) | 39.14 | 36.64 – 44.29 | <0.01 |
| Councils*FHP | -6.93 | -6.51- -7.45 | <0.01 |
| Environmental Sanitation Council | -0.42 | -0.20 – -0.88 | 0.35 |
| FHP Coverage | 0.21 | 0.02 – 0.43 | 0.41 |
| Local State Capacity | -0.90 | -0.64 – -1.14 | 0.02 |
| Mayoral Vote Share | -0.28 | 0.19 – -0.59 | 0.32 |
| Left Mayor | 0.18 | 0.01 – 0.47 | 0.29 |
| Health Spending (per capita, logged) | -0.76 | -0.54 – -1.12 | <0.01 |
| Observations | 5381 | | |
| Wald $Chi^2$ (6) | 224.85 | | |
| Prob> $Chi^2$ | 0.00 | | |
| $R^2$ | 0.40 | | |

Table S3 Negative Binomial Regression with Interactions between councils and local state capacity

| | Municipal Zika Cases per 100,000 Residents | | |
|---|---|---|---|
| Predictors | Estimates | CI | p |
| (Intercept) | 41.28 | 36.97 – 48.19 | <0.01 |
| Councils*Capacity | -8.14 | -7.92- -8.39 | <0.01 |
| Environmental Sanitation Council | -0.22 | -0.03 – -0.52 | 0.34 |
| FHP Coverage | -0.66 | -0.27 – -0.85 | 0.03 |
| Local State Capacity | -0.05 | 0.12 – -0.19 | 0.20 |
| Mayoral Vote Share | -0.26 | 0.18 – -56 | 0.31 |
| Left Mayor | 0.35 | 0.10 – 0.54 | 0.28 |
| Health Spending (per capita, logged) | -0.93 | -0.74 – -1.22 | <0.01 |
| Observations | 5365 | | |
| Wald $Chi^2$ (6) | 244.92 | | |
| Prob> $Chi^2$ | 0.00 | | |
| $R^2$ | 0.36 | | |

Table S4 OLS Regression with Interactions between councils and local state capacity

| | | Municipal Zika Cases per 100,000 Residents | |
|---|---|---|---|
| Predictors | Estimates | CI | p |
| (Intercept) | 38.20 | 34.17 – 46.60 | <0.01 |
| Councils*Capacity | -7.99 | -7.64- -8.25 | <0.01 |
| Environmental Sanitation Council | -0.18 | -0.03 – -0.36 | 0.39 |
| FHP Coverage | 0.71 | 0.42 – 0.93 | 0.38 |
| Local State Capacity | -0.11 | 0.16 – -0.40 | 0.31 |
| Mayoral Vote Share | -0.30 | -0.05 – -0.57 | 0.28 |
| Left Mayor | 0.15 | 0.03 – 0.39 | 0.18 |
| Health Spending (per capita, logged) | -0.80 | -0.68 – -0.92 | <0.01 |
| Observations | 5370 | | |
| Wald $Chi^2$ (6) | 218.67 | | |
| Prob> $Chi^2$ | 0.00 | | |
| $R^2$ | 0.39 | | |

Table S5 Negative Binomial Regression with Interactions between councils and health spending

| | | Municipal Zika Cases per 100,000 Residents | |
|---|---|---|---|
| Predictors | Estimates | CI | p |
| (Intercept) | 36.55 | 33.29 – 42.34 | <0.01 |
| Councils*Spending | -7.64 | -7.14- -8.10 | <0.01 |
| Environmental Sanitation Council | -0.29 | -0.01 – -0.62 | 0.40 |
| FHP Coverage | -0.86 | -0.19 – -0.94 | 0.01 |
| Local State Capacity | -0.85 | 0.21 – -1.45 | 0.33 |
| Mayoral Vote Share | -0.26 | 0.18 – -56 | 0.31 |
| Left Mayor | 0.14 | 0.01 – 0.31 | 0.18 |
| Health Spending (per capita, logged) | -0.11 | 0.23 – -0.44 | 0.16 |
| Observations | 5379 | | |
| Wald $Chi^2$ (6) | 248.15 | | |
| Prob> $Chi^2$ | 0.00 | | |
| $R^2$ | 0.43 | | |

Table S6 OLS Regression with Interactions between councils and health spending

| | | Municipal Zika Cases per 100,000 Residents | |
|---|---|---|---|
| Predictors | Estimates | CI | p |
| (Intercept) | 36.15 | 32.93 – 41.50 | <0.01 |
| Councils*Spending | -7.63 | -7.10- -8.03 | <0.01 |
| Environmental Sanitation Council | -0.25 | -0.06 – -0.47 | 0.31 |
| FHP Coverage | -0.74 | -0.52 – -0.91 | <0.01 |
| Local State Capacity | -0.49 | -0.13 – -0.61 | 0.04 |
| Mayoral Vote Share | -0.30 | -0.05 – -0.57 | 0.28 |
| Left Mayor | -0.22 | -0.06 – -0.43 | 0.19 |
| Health Spending (per capita, logged) | -0.09 | 0.27 – -0.35 | 0.26 |
| Observations | 5371 | | |
| Wald $Chi^2$ (6) | 223.52 | | |
| Prob> $Chi^2$ | 0.00 | | |
| $R^2$ | 0.40 | | |
